# Transposable element persistence via potential genome-level ecosystem engineering

**DOI:** 10.1186/s12864-020-6763-1

**Published:** 2020-05-19

**Authors:** Stefan C. Kremer, Stefan Linquist, Brent Saylor, Tyler A. Elliott, T. Ryan Gregory, Karl Cottenie

**Affiliations:** 1 School of Computer Science, University of Guelph, Guelph, ON N1G 2W1 Canada; 2 Department of Philosophy, University of Guelph, Guelph, ON N1G 2W1 Canada; 3 Department of Integrative Biology, University of Guelph, Guelph, ON N1G 2W1 Canada; 4 Centre for Biodiversity Genomics, University of Guelph, Guelph, ON N1G 2W1 Canada

**Keywords:** Ecosystem engineering, Transposon ecology, C-value paradox, Transposon accumulation, Junk DNA, Genome-level ecology

## Abstract

**Background:**

The nuclear genomes of eukaryotes vary enormously in size, with much of this variability attributable to differential accumulation of transposable elements (TEs). To date, the precise evolutionary and ecological conditions influencing TE accumulation remain poorly understood. Most previous attempts to identify these conditions have focused on evolutionary processes occurring at the host organism level, whereas we explore a TE ecology explanation.

**Results:**

As an alternative (or additional) hypothesis, we propose that ecological mechanisms occurring within the host cell may contribute to patterns of TE accumulation. To test this idea, we conducted a series of experiments using a simulated asexual TE/host system. Each experiment tracked the accumulation rate for a given type of TE within a particular host genome. TEs in this system had a net deleterious effect on host fitness, which did not change over the course of experiments. As one might expect, in the majority of experiments TEs were either purged from the genome or drove the host population to extinction. However, in an intriguing handful of cases, TEs co-existed with hosts and accumulated to very large numbers. This tended to occur when TEs achieved a stable density relative to non-TE sequences in the genome (as opposed to reaching any particular absolute number). In our model, the only way to maintain a stable density was for TEs to generate new, inactive copies at a rate that balanced with the production of active (replicating) copies.

**Conclusions:**

From a TE ecology perspective, we suggest this could be interpreted as a case of ecosystem engineering within the genome, where TEs persist by creating their own “habitat”.

## Background

Transposable elements (TEs) make up a large fraction of all but the most diminutive eukaryotic genomes and are the most prominent contributors to the enormous variability in nuclear genome size [1]. They also make up a small but significant portion of many prokaryotic genomes [2, 3]. Explaining both their extraordinary abundance and their differential representation among taxa has been a central challenge in evolutionary genetics for several decades. The earliest interpretation, and one that persists in many modern discussions, is that the abundance of TEs reflects real or potential functions that benefit the host organism in which they reside. Such proposed functions have included gene regulation (e.g. [4, 5]), buffering against mutations [6], causing mutations and generating new variation [7], and various other roles. Indeed, there has long been an assumption that TEs must impart some fitness benefit to organisms, or else they would have been pruned by natural selection long ago. Adaptationist interpretations of TEs and other non-genic DNA sequences remain prominent in some areas of genomics [8].

Nearly 40 years ago, the classic “selfish DNA” papers sought to characterize sequences such as TEs as biological entities with properties that could promote their accumulation even at the expense of host fitness [9, 10]. These early papers noted that, on occasion, initially selfish DNA elements could be co-opted into functional roles at the organism level. However, as genomic parasites, such functions were not necessary to account for their accumulation and persistence within genomes. More recently, the relationship between particular TEs and their hosts has been understood to vary along a continuum from strict parasitism to commensalism to mutualism [11]. Under this view, the impacts of TEs on their hosts can range from beneficial to neutral (or nearly so) to varying degrees of deleteriousness. There is a variety of specific scenarios that would allow TEs to accumulate and remain active. It is important to note that the categories, below, apply to individual TE families (or even individual copies or lineages within a family) and not to genomes as a whole; a given genome may be home to diverse TEs that occupy any number of these categories.

In very general terms, these scenarios can be summarized as follows:

1. TE insertions are beneficial and accumulate through positive selection on hosts. If individual TE insertions confer a net fitness benefit to the hosts that carry them, then these can accumulate through positive directional selection at the host level. TE accumulation by host-level selection will be particularly likely to occur if both the beneficial impact and host population size are large. That TEs begin as beneficial without any coevolution with or cooption by hosts may be a rather infrequent occurrence, but if it does occur then it is obvious that they will accumulate within a population by positive selection on hosts. In any case, adaptationist interpretations of TEs implicitly assume that the most common reason for accumulation of TEs is that they conferred a significant net benefit to their hosts [12–14].
2. TEs are (nearly) neutral and accumulate by genetic drift among hosts. If, on average, TE insertions have little or no impact on host fitness, then the primary means by which they can accumulate within genomes will be through genetic drift or mutation pressure (or both). In the case of genetic drift, individual TE insertions that are neutral (or at most mildly deleterious) may increase in frequency in the host population by chance, especially if the host population size is small [15]. TEs with minimal impacts on host fitness may also become more abundant if their rate of duplicative transposition exceeds the rate of loss by chance (e.g., through deletion bias [16];).
3. TEs (co-)evolve to become less deleterious and then accumulate through host-level evolutionary processes. If TE insertions are modestly, but more than very slightly, deleterious then they will be less likely to accumulate through genetic drift even in smaller host populations and are unlikely to persist long term, especially in large host populations in which negative directional selection exerts a stronger effect. Nonetheless, growth in TE numbers could still occur if the elements evolve properties that make them less detrimental to their hosts, such as through reduced replication rates or acquiring preferences for insertion sites where adverse insertional effects are minimal [12, 17]. Alternatively (or in addition), hosts may evolve mechanisms to reduce the deleterious effects of TE insertions such as limiting the rate of transposition. (For TEs to continue to accumulate, this can’t involve complete silencing or deletion of TE copies by the host). Evolutionary changes such as these at the TE and/or host level would shift the scenario to category 2 (above) where TE insertions would evolve as neutral alleles. It is also possible that changes in the TEs and/hosts could lead to the co-option of that TE into a host-level function, thereby shifting the relationship to category 1 (above).
4. TEs are sexually transmitted genomic parasites: TE insertions are significantly deleterious but spread via recombination. Even under a scenario in which TE insertions tend to be quite detrimental to host fitness, there can nonetheless be an accumulation of active TE copies if they spread more quickly than they can be deleted from the host population [18]. In this case, the relationship between TEs and hosts would reflect the dynamics typical of a virulent but non-fatal pathogen (i.e., TEs spread more quickly than they can be deleted) to which hosts are not strongly immune (i.e., host genomes are not able to effectively delete or silence the active TEs). If the fitness costs to the host are significant, then this is likely to require an effective means for TEs to spread within the host population, such as recombination. Indeed, it has long been recognized that TEs could spread more effectively in sexual versus asexual species, especially if they are detrimental to host fitness [19]. In other words, successful TEs in this category may usually be “sexually transmitted nuclear parasites” [19].
5. TEs can spread among species: TE insertions are severely deleterious but spread quickly and horizontally to new hosts. TEs whose activity impose very serious costs to their hosts would, under most conditions, be expected to be lost from the population, as individuals containing them are strongly selected against at the organism level. For TEs in this category to persist for more than a few host generations, let alone accumulate to large numbers, there must be a mechanism for them to spread to new hosts at a rate that counteracts the reduced reproductive success of individuals that contain them. To wit, they must be able to spread horizontally among hosts. This requirement would be especially strong for TEs that inhabit asexual hosts where, in the absence of recombination, they would otherwise be confined to vertical transmission pathways.

To summarize, mechanisms theoretically exist by which TEs can become abundant and remain active regardless of the nature of their initial relationship to host fitness. In some cases, as when TE insertions exert a beneficial effect at the host level, their accumulation is both likely and expected, especially in large host populations. Neutral or nearly neutral TE insertions can also accumulate, but this may require that host populations be relatively small. Deleterious TE insertions are not doomed to be lost, even if their impacts are rather severe, so long as they and/or their hosts can evolve in such a way that these adverse effects are mitigated or the TEs are able to spread to new genomes at a rate exceeding their loss by purifying selection among hosts. In the latter case, it is likely that additional mechanisms of spread such as recombination and/or horizontal transfer are required.

What happens if none of the above scenarios applies? It may be rare for none of these conditions to apply at all in a eukaryotic population, but because recombination, horizontal transfer, and/or mechanisms of co-evolution with hosts are known to be significant in affecting the accumulation of TEs, it is necessary to control for these in order to assess whether any other, less well established, factors also act on TEs independently. This is conceptually similar to having idealized but unrealistic null conditions in classical population genetics models (there are no infinitely large populations, for example). In both Zeyl et al. [20] and Bast et al. [21] experimental populations of yeast only accumulated TEs in sexual rather than asexual populations, in contrast to what we found under some parameter conditions in our simulations. Simulations by Bast et al. [21], using a modified model from Dolgin and Charlesworth [22], suggested the decrease in TE load in asexual populations occured via increased rates of element deletion from the genome over time. By contrast, we ask, can TEs accumulate in an asexual host with no horizontal transmission, even if they remain deleterious, and if neither they nor their hosts can evolve to change the nature of their relationship? In this study, we sought to answer this question by developing an idealized in silico simulation in which TE insertions into genes have serious negative effects on host fitness (ruling out category 1 and 2); TEs could not evolve insertion site preferences and hosts could not evolve TE-regulating mechanisms (eliminating category 3); the hosts reproduce asexually and there is no mechanism for horizontal transfer (excluding categories 4 and 5). Elsewhere, this has been described as genome-level ecology by Linquist et al. [23]. Briefly, the term “TE ecology” refers to changes in TE abundance and distribution that do not involve changes in TE sequence -- that is, based on interactions between TEs and other TEs, or with the host genome, but not involving evolution of TE or host.

Based on previous explanations (both theoretical considerations and observations of TE profiles in sexual vs. asexual taxa [23–25]), we would expect TEs to be unlikely to persist under such conditions as either they will be unable to spread, or they will be eliminated from the population by host-level selection, or the host populations will be driven to extinction by the accumulation of detrimental mutations with no means of restoring undamaged collections of genes through recombination (i.e., Muller’s ratchet). Intriguingly, our simulation indicates that TEs can, in fact, accumulate and persist – even if they are detrimental, and without coevolution, recombination, or horizontal transfer -- as long as certain conditions are met, including relatively low TE death rates, low TE progeny rates, low mutation rates, high mutation effects.

## Results

**Fig. 1:**
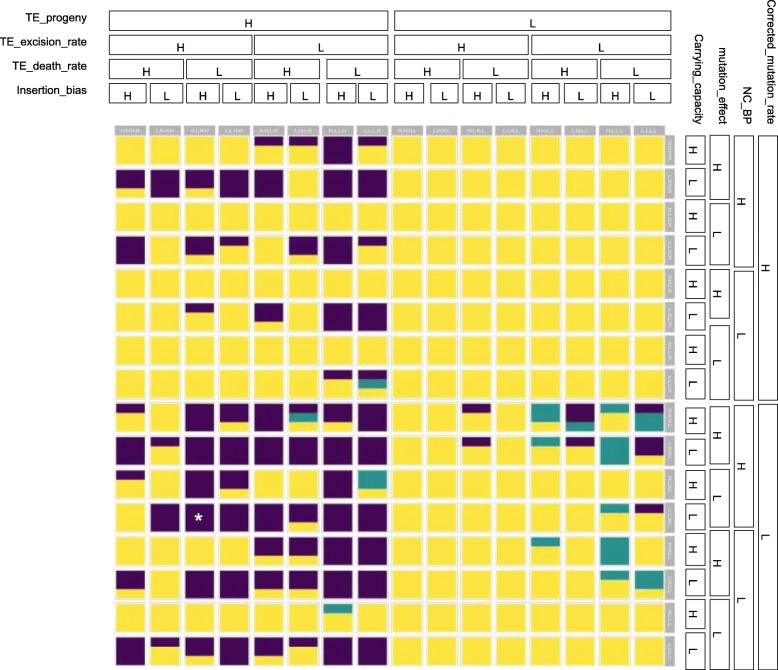
TE accumulation outcomes for the different simulations. The proportion of the three different accumulation patterns, with host extinction purple, TE accumulation teal, and TE extinction yellow, for the different combinations of simulation parameters. For illustration purposes, the ROWS are unique combinations of 4 host properties (Carrying_capacity, mutation_effect, NC_BP, Corrected_mutation_rate), and the COLUMNS unique combinations of 4 TE properties (Insertion_bias, TE_death_rate, TE_excision_rate, and TE_progeny). The column headings indicate whether it that column is either the high (H) or low (L) condition for these 4 experimental variables, in the order outlined above. The row headings follow the same format

We observed three broad classes of outcomes of our models (Fig. 1) as we varied the parameters described above: (1) TE extinction, in which no active TEs remain. There are three potential mechanisms for this: First, TEs become inactive via mutation. Second, active TEs are excised but do not reinsert in the genome. Third, hosts with active TEs die while hosts with inactive TEs persist. (2) Host extinction, in which all hosts in the population die. (3) TE accumulation, in which both TEs and hosts persist for the duration of the simulation. In the figure, the colouring of each square represents the outcomes of the three runs of a particular experimental design combination of Low/High variations. TE extinction is represented by yellow, host extinction by purple, and TE accumulation by teal. Squares with uniform colour represent experimental design combinations in which all three runs provided consistent outcomes, while multi-coloured squares are combinations with several different outcomes. A completely yellow box indicates that all three experimental replicates lead to extinction of the TEs. The location of a square within the array indicates the particular experimental design combination being reported.

Rows correspond to the Low and High variations of the Carrying_capacity, Mutation_effect, NC_BP, and Corrected_mutation_rate parameters, as respectively indicated in the row labels at the right of the figure. Columns correspond to the Low and High variations of the Insertion_bias, TE_death_rate, TE_excision_rate, and TE_progeny parameters, as respectively indicated in the column headers. For example, the cell marked “*”, because it is in the row labelled “L.L.H.L.” corresponds to the three runs in which Carrying_capacity was Low, mutation_effect was Low, NC_BP was High, and Corrected_mutation_rate was Low. Similarly, because that cell lies in the column labelled “H.L.H.H.”, it corresponds to the runs in which Insertion_bias was High, TE_death_rate was Low, TE_excision_rate was High, and TE_progeny was High.

In 575 experiments (or 75%), the TEs went extinct (indicated by yellow). In 169 experiments (or 22%), the rapid TE accumulation resulted in the host population extinction, which stopped the simulation (indicated by purple). And only in 23 experiments or (3% of all experiments) did the TEs accumulate without leading to host extinction (indicated by teal). It is this third result that represents a novel discovery.

In this analysis, we were interested only in whether or not it is possible for TEs to accumulate and persist under the conditions outlined in our introduction. When each unique experimental design combination was replicated three times, we only observed two experimental design combinations (full teal square) with a consistent accumulation pattern in the three replicates (1: Low Carrying_capacity, High mutation_effect, High NC_BP, Low Corrected_mutation_rate, High Insertion_bias, Low TE_death_rate, Low TE_excision_rate, Low TE_progeny and 2: High Carrying_capacity, High mutation_effect, Low NC_BP, Low Corrected_mutation_rate, High Insertion_bias, Low TE_death_rate, Low TE_excision_rate, Low TE_progeny). In all other experimental design combinations with a TE accumulation pattern, there was at least one replicate that resulted in either a host extinction or TE extinction.

The 23 experiments in which some TE accumulation patterns were observed occurred in 15 unique experimental design combinations. Table 1 summarizes the values (high/low) of the 8 experimental design parameters, Carrying_capacity, Mutation_effect, NC_BP, Corrected_mutation_rate, Insertion_bias, TE_death_rate, TE_excision_rate, and TE_progeny, for each of the experiments resulting in TE accumulation.

**Table 1:** Variable values where TEs Accumulate

Variable:	Carrying_capacity	Mutation_effect	NC_BP	Corrected_mutation_rate	Insertion_bias	TE_death_rate	TE_excision_rate	TE_progeny
**How often “H”:**	14	18	14	1	14	6	0	5
**How often “L”:**	9	5	9	22	9	17	23	18

We tested whether these 8 parameters could predict TE accumulation with a binomial ANOVA. Because of all the 0 s in this analysis (745 runs without TE accumulation vs. 23 with TE accumulation), we could only test for the main effects parameter effects (as opposed to a full-factorial ANOVA). Of the 8 potential main effects, 4 were significant: TE_death_rate (*P* = 0.01), TE_progeny (*P* = 0.004), Corrected_mutation_rate (*P* < 0.001), and Mutation_effect (*P* = 0.005).

**Fig. 2:**
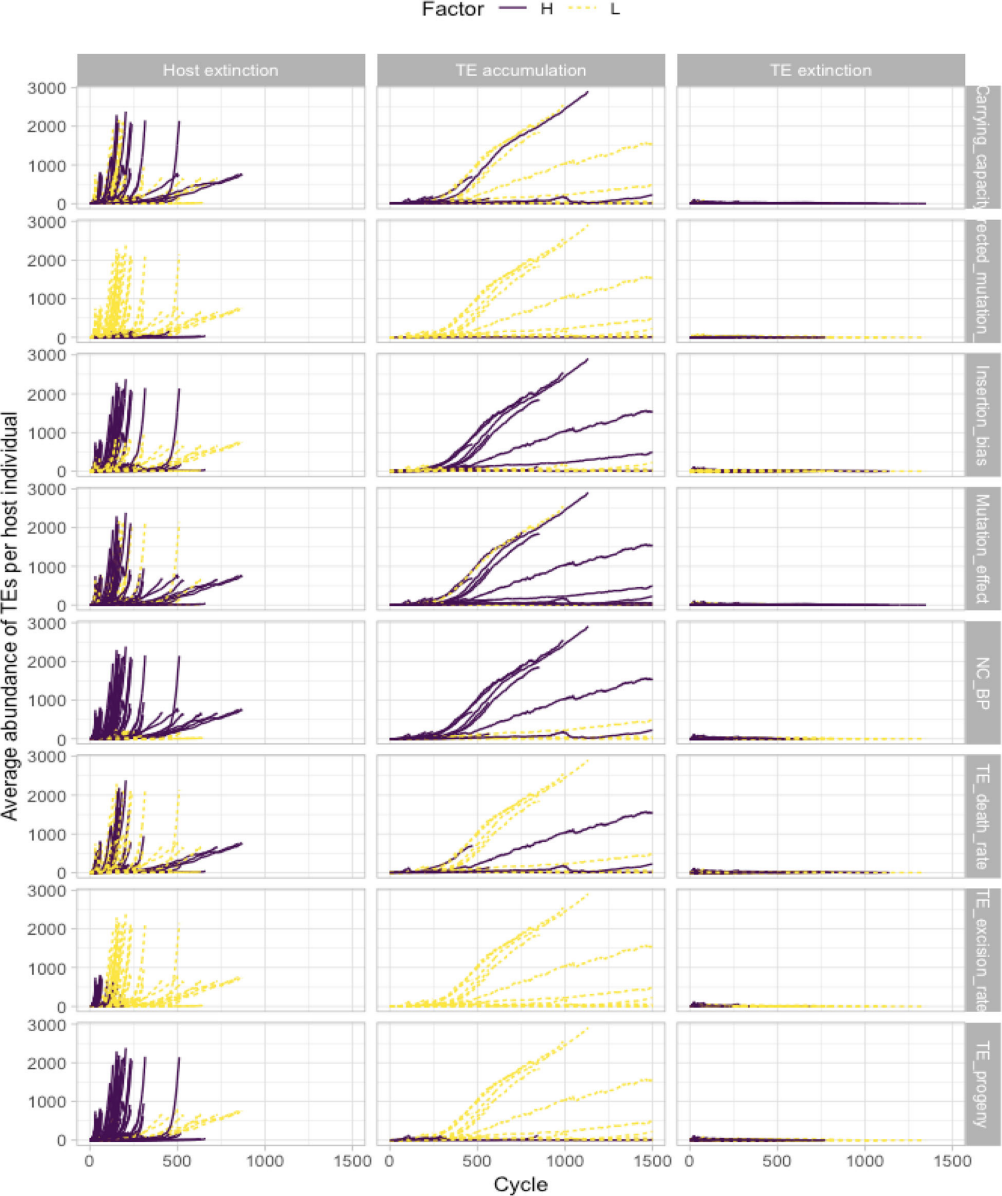
Active TE accumulation patterns for the different simulations. Average abundance of TEs per host individual is plotted as a function of the cycle time. The three different accumulation patterns (host extinction, TE accumulation, and TE extinction) are plotted in different columns. Specifically, we plotted all 575 simulations which led to host extinction in the first column, and all 23 simulations which led to TE accumulation in the second column, and all 169 simulations which led to the TE extinction in the third column. The different colours and line types indicate whether it was the high (purple, solid line) or low (yellow, dashed line) condition that was associated with the accumulation pattern for each variable, and in fact, the only difference between the rows is the colouring of the lines. Simulations in column 2 which seem to end prematurely reached the maximum 72 h time limit. We did not increase the limit because the time taken per cycle was increasing exponentially

Next, we investigated the accumulation of TEs over time with respect to each of the three possible outcomes, focussing on the effect of one of the variable parameters at a time. In Fig. 2, each row represents the variable parameter of interest. In each row, the first graph shows the accumulation of TEs for all experiments that ended in host extinction, while the second graph shows the accumulation for those experiments that ended in TE accumulation and the last graph shows the experiments that ended in TE extinction. Each line represents the average number of active TEs across all the hosts in the population for one experiment. Purple lines are for those experiments where the parameter of interest was set to High, while yellow lines represent the parameter of interest in the Low condition. We noticed that in the host extinction outcomes, the TEs often seemed to exhibit exponential accumulation, while in the TE accumulation outcomes TE numbers seem to grow more steadily, and in the TE extinction outcomes TEs never accumulated to significant numbers.

**Fig. 3:**
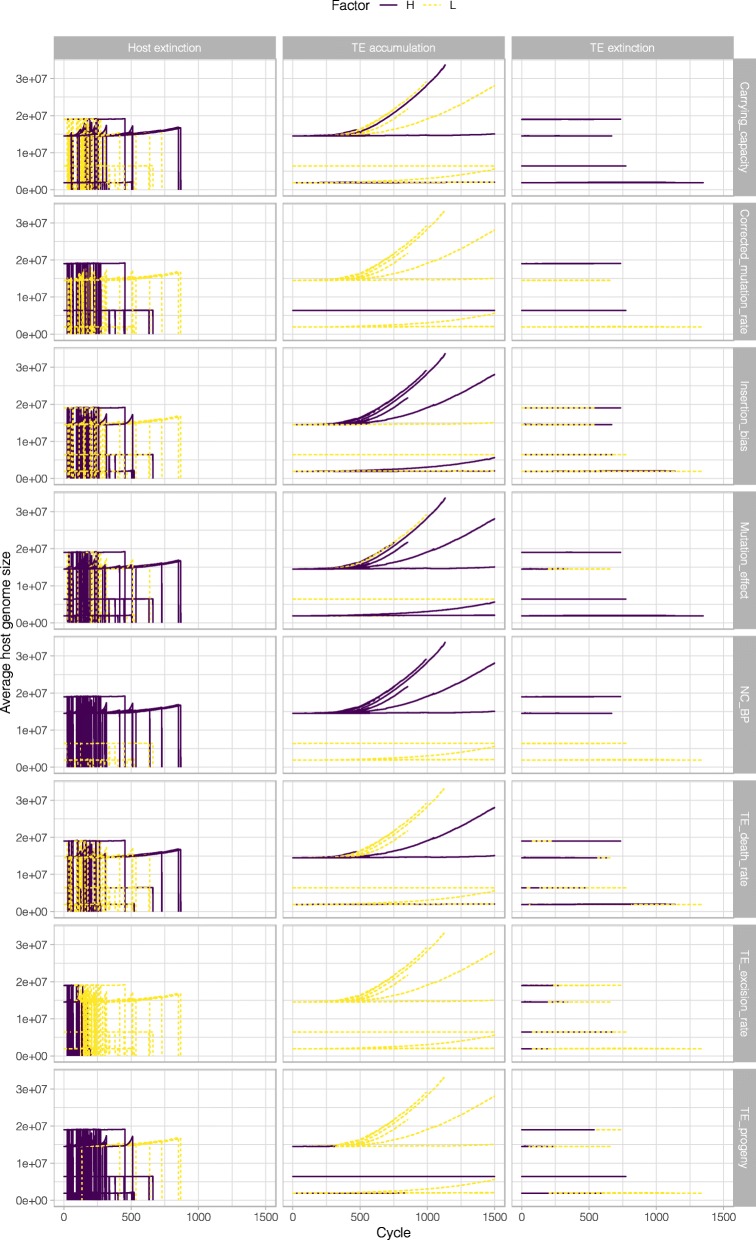
Average host genome size for the different simulations. Average host genome size is plotted as a function of the cycle time. The three different accumulation patterns (host extinction, TE accumulation, and TE extinction) are plotted in different columns. The different colours and line types indicate whether it was the high (or low) condition was associated with the accumulation pattern for each variable (or rows of plots)

We also tracked the average genome size over time, as depicted in Fig. 3. In our simulation, TEs can become inactive either when a mutation within the TE causes it to become incapable of transposition (this is governed by the TE_death_rate, see Methods, c. Simulation, Step 3, below), or if a second TE inserts itself into the space occupied by the former TE (see Methods, c. Simulation, Step 6, below). This figure also approximates the rate of inactive TE growth, since the only cause for additional genome size (beyond the initial non-coding base pairs, NC_BP) in our model is the accumulation of TEs and the growth of the number of inactive TEs dominates the growth of the active TEs.

**Fig. 4:**
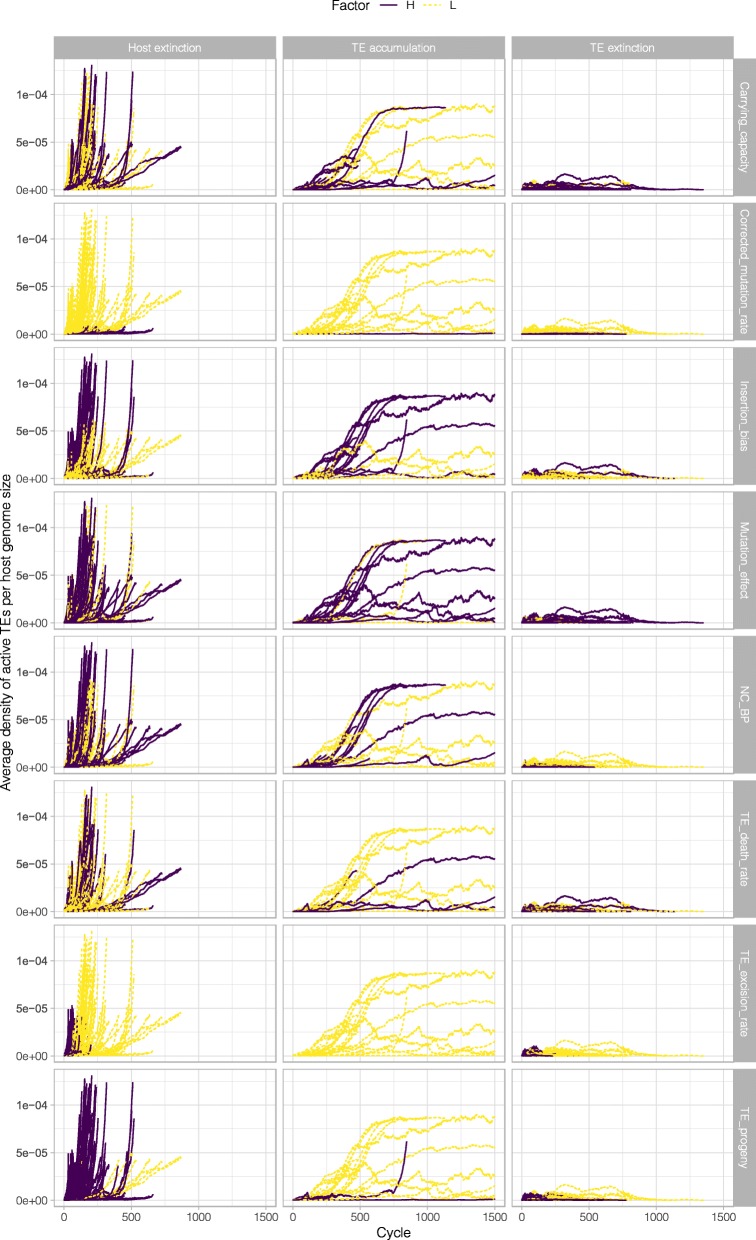
TE accumulation patterns for the different simulations. Average density of TEs per host genome size is plotted as a function of the cycle time. The three different accumulation patterns (host extinction, TE accumulation, and TE extinction) are plotted in different columns. The different colours and line types indicate whether it was the high (or low) condition was associated with the accumulation pattern for each variable (or rows of plots)

Finally, since Fig. 2 suggested that the difference between the TE accumulation versus the host extinction pattern could be caused by the speed of TE accumulation (high initial TE accumulation leading to host extinction), we plotted TE numbers also as densities by standardizing average TE abundances per host genome by genome size (Fig. 4). Figure 4 depicts the ratio of Fig. 2 to Fig. 3.

We noticed in Fig. 4 that the TE accumulation outcomes were characterized by TEs reaching a density plateau. Since TEs continue to accumulate even after they have reached a density plateau, and there is no DNA deletion in the model and there is no DNA addition other than via TE addition, the insertion of active TEs is balanced by a corresponding increase of inactive TEs.

## Discussion

We developed a model in which TEs are deleterious, do not evolve, and cannot transfer horizontally between hosts or to other species. This model explicitly violates the known explanations for the persistence of TEs, namely:

1. *TE insertions are beneficial.*
2. *TE insertions are (nearly) neutral.*
3. *TE insertions are somewhat deleterious (but evolve to become nearly neutral or beneficial).*
4. *TE insertions are significantly deleterious but spread* via *recombination or horizontal transfer.*
5. *TE insertions are severely deleterious but spread quickly and horizontally to new hosts.*

Thus, our expectation was to find no instances of TE accumulation. In fact, we found that TEs do accumulate sometimes, and more frequently under certain assumptions.

Why do TEs accumulate more rapidly in some genomes than others? The dominant view is that TE accumulation is driven by selection among hosts, or they accumulate when host populations experience drift, or that hosts genomes are in a balanced state of insertion/deletion. All three of these hypotheses predict that TEs cannot accumulate indefinitely so long as the host population is under strong selection and provided that they remain largely deleterious. Our model tested this prediction under a range of four different “TE ecological” parameters within the host (Carrying_capacity, Mutation_effect, NC_BP, Corrected_mutation_rate), and for four different TE parameters (Insertion_bias, TE_death_rate, TE_excision_rate, and TE_progeny), each under high and low conditions. The majority of these simulations bore out the received theoretical expectation of no accumulation of TEs that are on average deleterious (see Insertion_effect). TEs were purified from the genome in 75% of the cases, and they drove the host population extinct in another 22% of the conditions. However, there remained an intriguing handful of cases where, even under such selectively unfavourable conditions, TEs attained potentially high abundances. This occurred without the benefit of co-evolution or drift. Thus, contrary to received wisdom, it is theoretically possible for active TEs to achieve high abundances within a genome without becoming “domesticated” [26] and despite strong selection for their removal.

Interestingly, there was no single TE property, nor any particular host property, that reliably generated this pattern (see Fig. 1). It is noteworthy that under both conditions where TEs reliably reached high abundances, insertion rates (excision rate times progeny) were relatively low. Every insertion has the potential of hitting a gene, decreasing host fitness and thereby being selected against. This finding can be compared to the longstanding expectation that TEs must exhibit “self-restraint” in order to persist [27]. Our analysis builds upon this conclusion by providing a finer grained account of what self-restraint amounts to. Specifically, we note that reduced insertion rate is not sufficient for TE accumulation; it must occur in conjunction with particular “environmental” conditions within the host.

Our findings can also be compared to those of Le Rouzic and Capy [28], who modeled the invasion of a TE into a sexually reproducing host environment. They observed that initial colonization requires a high rate of replication and insertion. However, they note that an overly aggressive replication rate is ultimately self-defeating. Eventually, as the TE population reaches a critical level, the host population is driven to extinction. Le Rouzic and Capy [28] proposed that a potential solution to this problem is for the rate of TE replication to be carefully regulated. During the colonization phase, TEs would undergo a burst of replication activity; but after reaching a certain threshold, they would have to enter a phase of relative dormancy. Our model exhibited some of the same behaviour, in that TEs tended to either be purged from the genome or else they caused host extinction. However, we demonstrated that it is possible for TEs to persist and reach very high abundances without the need to attenuate replication.

How exactly did TEs manage to attain such high abundances in our model without driving the host population extinct? Figure 4 suggests an intriguing answer. Although TE abundances continued to climb in these cases, TE density within the genome leveled off at a certain point. Perhaps the most important lesson to be drawn from this study is that TE density, not absolute abundance, is the more important factor influencing TE persistence. Our fourth figure measures the density of TEs which is equivalent to the ratio of live to dead TEs as the genome size increases beyond the initial genome size.

As the abundance of TEs within a genome increases, the only way to stabilize density is by increasing the amount of non-mobile DNA. In nature, this could be achieved by a number of different mechanisms. For instance, chromosome duplication might be an efficient way to supply new real estate for a growing population of TEs. However, in our model the only available source of new DNA was supplied by the TEs themselves. We think that this suggests an interesting new mechanism by which TEs might accumulate more generally. Ecologists have long recognized that organisms sometimes manage to persist in an ecosystem by generating their own habitat. This phenomenon is described as ecosystem engineering [29]. Extending this idea to TE ecology, active TEs are comparable to organisms and defunct copies of TEs represent additional habitat in the form of safe insertion sites for active TEs. Presumably, this process would require the creation of a certain number of inert copies for every additional active copy that is added to the TE population. A question to be explored in a future study concerns the precise proportions of active vs inert copies that would give rise to a stable density.

## Conclusions

We propose that the TE ecosystem engineering hypothesis identifies a distinct process that potentially contributes to variability in genome size among species. To be clear, we do not take ourselves to have provided evidence that, in nature, the capacity for TEs to accumulate and remain active in most eukaryotic genomes is due to TE ecosystem engineering. This is an important empirical question which we are currently some distance from being able to answer. Nonetheless, our contention is that this hypothesis offers a novel and important addition to the stock of candidate explanations for variability in C-value. In addition, our analysis also provides more specific information on what TE and host properties would be beneficial to evolve to result in stable accumulation of TEs.

This proposal can be seen as an extension of Selfish DNA theory, in that it views the transposable element as the focal unit of analysis, somewhat autonomous from the genome in which it resides. However, a longstanding challenge for Selfish DNA theory has been to explain the differential success of TEs among different species. We have proposed that, generally speaking, this question is best addressed by investigating the local “TE ecological” conditions that TEs confront within the cell [29–31]. Interestingly, our current findings identify a particular TE ecological mechanism that could enable TEs to reach extremely high abundances in nature. This is consistent with viewing TEs as “selfish” in that their reproductive success does not require them to evolve to become more intrinsically benign or even beneficial for the host. More generally, this demonstrates the benefit of taking a TE ecological approach for generating novel hypotheses about transposon dynamics.

## Methods

### Model description

We built a novel computer simulation of a population of host organisms supporting TEs which we used to conduct a number of experiments, each with different parameters. Previous software models did not allow us to test the scenario that we wanted to explore. Specifically, according to the model presented by Dolgin and Charlesworth [22], TEs are lost from the host genome if there is a mechanism for TE deletion, regardless of host population size. On the other hand, if there is no TE deletion possible, then either hosts will go extinct if their populations are small or there will be an equilibrium number of TEs that remain if host TE populations are very large. In their model, host fitness is inversely correlated with TE abundance in the genome. Our model, by contrast, does not consider very large (“infinite”) host populations and incorporates variable fitness consequences in two ways: 1) each individual insertion has an independent, probabilistic effect on host fitness, and 2) the fitness effects of TE insertions are influenced by the quantity of non-coding DNA present, as this serves as safe sites into which TEs can insert without interrupting a gene. Their model did not include the inactivation of elements, nor the fact that elements could sometimes create beneficial mutations for the host, nor that genome size could vary via the amount of non-coding DNA present. Dolgin and Charlesworth [22] do briefly mention a scenario where there was runaway element copy number increase, but it is not detailed in the paper, nor the conditions under which it occurred. One thing that we wanted to observe is the effect of TEs inserting into genic and non-genic regions under different conditions. To achieve this objective, we explicitly tracked the location of every gene and every TE. We simulated the excision and reinsertion of TEs by generating new insertion locations of TEs according to two different probability distributions (see details below). During many of our simulations, the number of TEs grew to several thousand. Together with hundreds or thousands of genes, spread across populations of tens to hundreds of individuals, this involved individually tracking the location of up to millions of individual elements (TEs and genes). To make the model computationally practical under these conditions, we wanted to include as few biologically relevant parameters as possible. As described further below, we selected our parameters based on prokaryotic hosts where the most experimental information on TE properties was available. Our primary objective with these simulations was to develop a model that was as realistic as possible (whose parameters are consistent with biological observations) where TEs accumulate. In the description below, the parameters that can be specified by the program’s operator are given in parenthesized, italic letters.

### Initialization

Each experiment was initialized by creating a single host organism (and then cloning it to create an initial population). The host organism is single celled, asexual and has a single linear chromosome.

The original host organism is created with a specified number of base-pairs of non-coding DNA (NC_DNA). After creating such an “empty” chromosome, genes were added by placing them at random positions within the chromosome. The number of genes added were varied and are detailed in the Section, “e. Varied Parameters”, below. In our model each gene was 1000 bp in length (adding a gene would increase the length of the chromosome by 1000 bp). Genes were inserted according to a specified, not necessarily uniform, probability distribution (Gene_Insertion_Distribution). This allowed us to create gene-rich regions within the chromosome (where the probability distribution has a high value) and gene-poor regions (where the probability distribution has a low value). After inserting a specified number of genes (Inital_genes), we added a single TE (in our simulation each TE was 1000 bp in length). Just as with the genes, we allowed the user to specify a non-uniform probability distribution (TE_Insertion_Distributuion), which allowed us to model favourable insertion regions that were potentially different from the gene rich regions. In addition to using the TE probability distribution to insert the progenitor TE for our simulation, we also re-use the same distribution for all other TE insertions (i.e., as TEs excised and re-inserted in different locations in the genome). Finally, the host organism is assigned an initial survival likelihood variable (which later plays a role in host selection) of 1. Then, the original host is cloned to produce a population of a specified size (Carrying_capacity).

### Simulation

At this point a simulation can commence. The simulation proceeds by cycles. During each cycle, the following steps occur:

1. Each host individual in the population is cloned (resulting in a population of double the original size).
2. Each host clone has a probability (Host_mutation_rate) of being mutated. Mutations can be mildly beneficial, neutral, mildly deleterious, or fatal. The effect of mutation is modelled by adjusting the individual’s survival likelihood variable according to a probability distribution (Host_mutation), where beneficial, neutral, and deleterious mutations increase, do not change, and decrease the fitness variable respectively. The strength of mildly beneficial and deleterious mutations is governed by a value (Mutation_effect). Fatal mutations set the survival likelihood variable to zero. These mutation effects do not include the activity of TEs which can insert into genes and have an additional effect on the fitness variable (see item 6., below).
3. Each transposable element in each host organism has a specified probability of becoming inactive (TE_death_rate). We have no mechanism for inactive TEs to return to activity.
4. Each transposable element in each host organism has a specified probability of excising (TE_excision_rate). In our model, we assume that only excised TEs can have progeny (i.e. not like a retro-transposons that can be copied without being excised first).
5. Each excised TE has a probability distribution over the number of copies of itself that are reinserted into the chromosome (TE_progeny). This probability distribution covers the case of zero TEs (the original TE is excised and lost), one TE (the original TE jumps to a new location), or greater than one TE (new TE copies are added to the chromosome). The location of all reinserted TEs are drawn from the TE insertion probability distribution (TE_Insertion_Distributuion).
6. If a reinserted TE’s new location is inside a gene, a probability distribution (Insertion_effect) governs what happens to the host. The effects can be deadly (sets survival likelihood to zero), mildly deleterious (reducing survival likelihood), neutral (survival likelihood unchanged), or mildly beneficial (increasing survival likelihood). Again, the strength of mildly beneficial and deleterious mutations is governed by a value (Mutation_effect).
7. If a reinserted TE’s new location is inside another TE, then the destination TE is rendered permanently inactive.
8. A survival probability is computed for each host. This probability is equal to the host’s survival likelihood, divided by the sum of all survival likelihoods, multiplied by the carrying capacity of the environment (Carrying_capacity). The normalization of this probability causes the mathematical expectation of the number of survivors to equal the carrying capacity (while in a given draw, it may be more or less).

The simulation runs through steps 1–8 for multiple cycles until either:

1. The maximum number of steps is reached.
2. There a no more active TEs in any of the hosts.
3. All hosts have died.

We did not run an experiment with no TEs because the selection mechanism in the model is designed to maintain hosts at carrying capacity so host extinction should not occur in the absence of TEs.

Below is a summary of the fixed parameters that we used in all of our simulations, followed by a summary of the parameters that we varied in our experiments.

### Fixed parameters

Our simulation has a number of parameters that can be set. For our experiments, we fixed some of these parameters based on reasonable estimates drawn from the literature. In many cases, these estimates were not available for a single biological system, so we had to collect estimates from a range of organisms. These are detailed below.

#### Cycle length

Rather than trying to simulate generations individually (which would have been prohibitively time consuming), we decided to use a higher granularity by making the simulation proceed in cycles. Each cycle represents 10^7^ (ten million) generations. This allowed us to accelerate the rates of mutation, TE insertions, etc. Based on bacterial generation times (15 mins to 24 h), each of our cycles could represent anywhere between 6000 and 300,000 years.

#### Gene length (Gene_length)

This parameter represents the length of a gene measured in base-pairs. We modelled all genes as having the same length and without introns. The value used for all of the experiments detailed below was 1000 bp. This value was based on a rounding of the average length of a prokaryotic gene, which is around 924 bp [32].

#### TE length (TE_length)

This parameter represents the length of a TE we modelled all TEs with the same length. The value used for all of the experiments was 1000 bp. Again, we used a rounded off value of the average length of an IS element, which is around 1200 bp [2].

#### Host mutation effect table (Host_mutation)

We used the following probability table to determine the effect of mutations on host fitness over the period of a cycle in a host organism, irrespective of TE transposition. The effect of TE transposition was explicitly handled using the Insertion_effect parameter, Table 2.

**Table 2:** Probabilities of host mutation effects

Effect	Lethal (survival likelihood = 0)	Mildly deleterious^a^	Neutral (survival likelihood unchanged)	Mildly beneficial^a^
Probability	40%	30%	15%	15%

^a^The degree of mildly deleterious/beneficial mutations were varied in our experiments and are therefore detailed in the “Varied Parameters” section, below

This set of parameters was complex to estimate. Based on the literature, we found that 30–40% of mutations are lethal, 2–30% of mutations are neutral mutations, and 0–15% are beneficial [33].

#### Insertion effect table (Insertion_effect)

We used the following probability table to determine the effect of a TE inserting into a gene on host fitness within the host organism, Table 3.

**Table 3:** Probabilities of TE insertion effects

Effect	Lethal (survival likelihood = 0)	Mildly deleterious^a^	Neutral (survival likelihood unchanged)	Mildly beneficial^a^
Probability	30%	20%	30%	20%

^a^The degree of mildly deleterious/beneficial mutations were varied in our experiments and are therefore detailed in the “Varied Parameters” section, below

It was difficult to find data on the effects of TE insertions, but we did find an indication that 40% of TE insertions resulted in negative selection [34].

### Varied parameters

In addition to the fixed parameters that we used above; we also used some parameters that we varied across experiments. These are detailed here. Each parameter was instantiated with one of two values during a particular experiment. The two values were selected to give a High rate of TE proliferation vs a Low rate of TE proliferation.

#### Probability of TEs becoming inactive (TE_death_rate)

Our model included a probability (TE_death_rate) with which TEs became inactive during Step 3 of each cycle. The probabilities used were 0.0005 (High) and 0.005 (Low). We based these values on the observation that *Alu* elements which are 10% or more divergent from their consensus sequence show a precipitous drop-off in activity [35, 36]. Given our previous decision that our TEs consisted of 1000 bp, we decided that TEs that were subjected to 100 point mutations would become inactive.

We decided to consider a point mutation rate in the order of one per 10^8^–10^9^ generations based on mutation rates reported by [37]. Converting this to cycles gives a few mutations per 10^1^–10^2^ cycles. To accumulate the 100 point mutations required to become inactive (previous paragraph), would thus take on the order of about 10^3^–10^4^ cycles. This implies that the probability of a TE becoming inactive during any cycle is in the tenths or thousandths of a percent. Within this range we selected 0.0005 and 0.005.

#### TE excision rate (TE_excision_rate)

During Step 4 of each cycle, TEs would excise with a variable rate. The probability of each TE excising was either 0.5 (High) or 0.1 (Low). This was based on the replicative transposition rate of 4 × 10^− 8^/element/generation reported in [38]. Converting this to cycles gives an excision rate of 0.4/element/cycle.

#### TE progeny distribution (TE_progeny)

After excising, the TEs would reinsert during Step 5 with one of two probability distributions. Note that in each distribution the probabilities sum to 100% and that the difference between the probability distributions lies in whether 0 (i.e. the excised TE is lost) or 3 TEs are reinserted with 15% probability. We were unable to find literature discussing the rate of reinsertion after a TE excised, so we set up our model to have a net positive reinsertion rate with one more aggressive and one less aggressive insertion distribution, Table 4.

**Table 4:** TE progeny distribution

(High)				
Number of TEs Inserted	0	1	2	3
Probability	0%	55%	30%	15%
(Low)				
Number of TEs Inserted	0	1	2	3
Probability	15%	55%	30%	0%

#### Corrected mutation rate: (Corrected_mutation_rate, a function of Initial_genes and Host_mutation_rate)

Another varied parameter is the number of genes found in each host organism Since the host organisms start as clones and there is no mechanism for the creation of new genes or destruction of genes, the number of genes remains constant throughout the simulation across all host organisms. Having a large number of genes should increase the likelihood of one of those genes mutating and as a result the host organism having an increased likelihood of experiencing a mutation. Our model uses a host mutation rate, which is the probability that the host’s genes will be changed rather than the probability of an individual gene being mutated. For this reason, when we used a larger number of genes (ten-fold increase) we also increased the host mutation rate (ten-fold). The initial chromosome had either 5000 genes and a corresponding host mutation rate of 0.3 (High), or 500 initial genes and with a corresponding host mutation rate of 0.03 (Low). These parameters were based on the work of Land et al. (2015) which reviewed 20 years of bacterial genome sequencing.

#### Amount of non-coding DNA (NC_BP)

In addition to the genes and the TEs, the genome of the hosts contained an amount of non-coding (NC) DNA. We used 14 million (High) and 1.4 million (Low) base pairs for this parameter.

Given that we used models with 500–5000 genes with each gene being 1000 base-pairs in length, we had ½ to 5 million coding base-pairs on our model. We found that [39] reported that 38–90% of the bacterial genome is coding, while [40] noted 56–96%. Based on these numbers, we used 14 million and 1.4 missing base-pairs of non-coding DNA in our model. Fourteen million base-pairs is also consistent with [41].

#### Magnitude of beneficial/deleterious mutation effects (Mutation_effect)

There are two causes of mutation in the model: natural mutation of the host’s genes over time, and the effect of TE insertion into a gene. In each of these cases, a probability distribution is used to determine the effect on the host organism’s survival likelihood: lethal, mildly deleterious, no change, mildly beneficial. These probability distributions for the two types of mutation have been described above in the “Fixed Parameters” section. One aspect of mutation that we did vary across experiments, was the magnitude of a beneficial or deleterious mutation. When a beneficial/deleterious mutation occurs, a random number between 0.0 and 1.0 is drawn from a uniform distribution, multiplied by the Mutation Effect (mutation_effect) parameter and added-to/subtracted-from the survival likelihood of the individual. The two Mutation Effects used were 0.1 (High) and 0.01 (Low).

We found a number of studies discussing the selection coefficients of TE insertions on the host ranging from 0.004 [42], to 0.01 [43] to 0.1 [44]. Additionally, [45, 46] found that deleterious mutations resulted in fitness reductions of the host of 10 and 7% (respectively) relative to the wild type. Based on these values we decided to implement an increase/reduction of 0.1 or 0.01 to the survival likelihood for beneficial/deleterious mutations.

#### Carrying capacity (Carrying_capacity)

The model allows large or small host population sizes which aren’t fixed, but trend to the specified carrying capacity (Carrying_capacity) of the host environment. We set the capacity to 300 (High) or 30 (Low) host individuals.

#### Insertion bias: insertion_bias, a function of gene rich areas and TE Insertion preferences (Gene_insertion_distribution, TE_insertion_distribution)

**Fig. 5:**
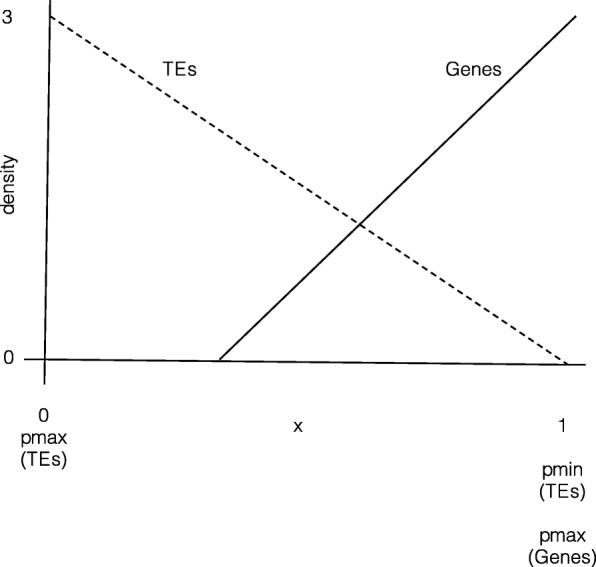
Gene and TE distributions

Our model allows us to simulate gene rich and gene poor areas of the chromosome and also to allow TEs to preferentially insert into different regions of the chromosome (e.g. gene poor regions). For our experiments, we decided to explore two scenarios. In the first (Low bias zone), we distributed genes randomly, but uniformly throughout the chromosome and we similarly allowed TEs to insert with uniform probability in all regions of the chromosome. In the second scenario (High bias, away from high gene density regions), we distributed genes preferentially at one end of the chromosome, while we allowed TEs to preferentially insert at the other end (Fig. 5). Note, that we are not suggesting that such a simple distribution accurately represents the distribution and preferential insertion of genes and TEs respectively in a real chromosome, but rather we wanted to explore a simulation with gene dense regions and preferential TE insertion in a simplified model. Note that despite preferring different regions, there is overlap in both scenarios, where genes lie and TEs insert.

### Experiments

Each experiment was allowed to run for up to 72 h on a high-performance computing cluster using a 2.1GHz processor and access to 100GB of RAM [47]. Due to the computational requirements of the simulation, the number of experiments that could be completed in a reasonable amount of time was limited. As can be seen above, there were 8 different Low/High variations used, resulting in 2^8^ = 256 experimental design combinations. Fortunately, since our main result is to show that certain outcomes are possible, we did not require many replications of the combinations (which would have been required if we were making statistical claims about frequencies or other measurements of quantitative effects). So, each design was run 3 times, for a total of 768 experiments.

## Data Availability

The software described below and used to generate the results described is available at: https://github.com/stefan-c-kremer/TE_World2. All datasets were generated from this software and can be regenerated by interested readers.

## Abbreviations

bp: Base pairs
SHARCNet: Shared Hierarchical Academic Research Computer Networks
TE: Transposable Element
